# Adsorption Modeling Based on Classical Density Functional Theory and PC-SAFT: Temperature Extrapolation and Fluid Transfer

**DOI:** 10.1021/acs.iecr.4c01395

**Published:** 2024-08-01

**Authors:** Fabian Mayer, Philipp Rehner, Jan Seiler, Johannes Schilling, Joachim Gross, André Bardow

**Affiliations:** † Energy & Process Systems Engineering, Department of Mechanical and Process Engineering, ETH Zurich, 8092 Zurich, Switzerland; ‡ Institute of Thermodynamics & Thermal Process Engineering, University of Stuttgart, 70569 Stuttgart, Germany

## Abstract

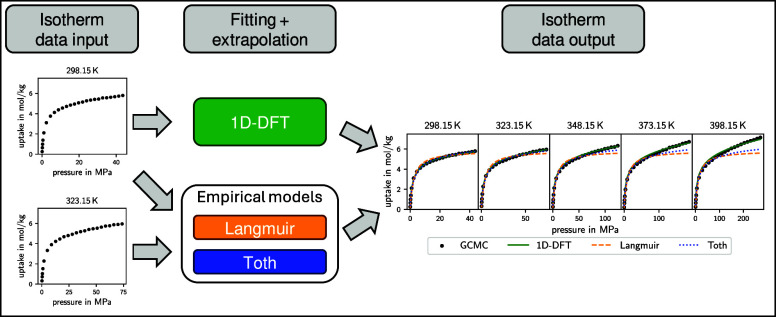

Adsorption is at the heart of many processes from gas separation to cooling. The design of adsorption-based processes requires equilibrium adsorption properties. However, data for adsorption equilibria are limited, and therefore, a model is desirable that uses as little data as possible for its parametrization, while allowing for data interpolation or even extrapolation. This work presents a physics-based model for adsorption isotherms and other equilibrium adsorption properties. The model is based on one-dimensional classical density functional theory (1D-DFT) and the perturbed-chain statistical associating fluid theory (PC-SAFT). The physical processes inside the pores are considered in a thermodynamically consistent approach that is computationally efficient. Once parametrized with a single isotherm, the model is able to extrapolate to other temperatures and outperforms the extrapolation capabilities of state-of-the-art models, such as the empirical isotherm models from Langmuir or Toth. Furthermore, standard combining rules can be used to transfer parameters adjusted to an adsorbent/fluid pair to other fluids. These features are demonstrated for the adsorption of N_2_, CH_4_, and CO_2_ in metal-organic frameworks. Thereby, the presented model can calculate temperature-dependent isotherms for various fluids by using data limited to a single isotherm as input.

## 1 Introduction

The efficient separation of gas mixtures is crucial for a transition to a sustainable chemical industry. A separation process of major current interest is CO_2_ capture from point sources and ambient air.^1−4^ Marco Mazzotti, to whom this special issue is dedicated, has been a pioneer in adsorption-based CO_2_ capture and is still a leader in the field, with contributions ranging from fundamentals^5−8^ to large-scale demonstration.^9−11^ Furthermore, adsorption is used for, e.g., biogas upgrading^12,13^ or air separation.^14,15^ Physical adsorption using solid porous materials is promising for gas separation because these materials can efficiently capture large amounts of gas and are easy to regenerate.^16^

The performance of adsorption-based processes depends on the solid material used as adsorbent and its interaction with the gaseous mixture involved, the adsorbate.^2,17^ Therefore, the design of adsorption processes depends on the thermodynamic properties of the adsorbent–adsorbate pairs. Important thermodynamic equilibrium properties are the enthalpy of adsorption and the uptake (also called loading) of the adsorbent–adsorbate pair for a given temperature and pressure,^18^ usually reported as adsorption isotherms.

Data for adsorption isotherms can be obtained through experiments.^19^ However, experiments are time-consuming and expensive.^20^ An enormous experimental effort is necessary to obtain adsorption isotherm data for a broad range of adsorbents, fluids, temperatures, and pressure levels.

The experimental effort could be reduced by predicting isotherms computationally, for which molecular simulations have developed into powerful tools.^21−23^ The two prevalent methods employed for molecular simulations are Molecular Dynamics (MD)^24,25^ simulations and Grand Canonical Monte Carlo (GCMC) simulations.^20,26^ However, despite the reduced time demand and costs compared to experiments, the computational effort of molecular simulations is still high for the prediction of isotherm data for various temperatures as well as a broad range of adsorbents and fluids that are relevant for process design.^27^

In addition, process simulations and design require simple, noise-free, and fast models for isotherm calculations. For this purpose, empirical isotherm models have been developed that simplify the physics inside the pores of the adsorbent. Overviews of empirical isotherm models are given by Do,^28^ Foo and Hameed,^29^ Alberti et al.,^30^ Wang and Guo,^31^ Chilev et al.,^32^ Hu et al.,^33^ and Serafin and Dziejarski.^34^ Empirical isotherm models are first parametrized to isotherm data from either experiments or molecular simulations. Afterward, the models can be used to estimate uptakes at pressures and temperatures that have not been measured experimentally or predicted by molecular simulations.

Empirical isotherm models can be characterized by the number of model parameters. The model parameters can be temperature-dependent. Typically, the temperature dependence of the parameters is described by simple relations, which introduce at least one additional model parameter for each temperature-dependent parameter. Thermodynamics imposes two conditions on isotherm models:^32,33^ (1) for low pressures, the model should reduce to Henry’s law, i.e., the amount of gas that is adsorbed in the porous medium is directly proportional to the partial pressure of the gas outside of the porous medium, and (2) for high pressures, the adsorbed amount reaches a plateau at its maximum value.

Two empirical isotherm models that are widely applied in literature are the Langmuir isotherm model^35^ and the Freundlich isotherm model.^36^ The assumptions of the Langmuir model are^28,32^ the adsorbent has a homogeneous surface, the adsorption energy over all adsorption sites is constant, and each adsorption site of the surface can only host one molecule, which implies that only monolayer adsorption is possible.

The Langmuir isotherm model is typically described by the following equations as presented by Do:^28^
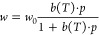
1with
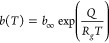
2

In eqs 1 and 2, *w* is the uptake, *T* the temperature, *p* the pressure, and *R*_*g*_ the ideal gas constant. The affinity parameter *b*(*T*) of the Langmuir model is temperature-dependent. The parameters *w*_0_, *b*_*∞*_, and *Q* are model parameters.

Despite the simplified assumptions of the Langmuir isotherm model, both conditions for a thermodynamic basis (at low and at high pressures) are met. Therefore, the model has been applied for a wide range of pressures, allowing it to be very beneficial to a range of fields within chemical science.^37^

The Freundlich isotherm model is not limited to monolayer adsorption and can describe heterogeneous surfaces. However, Henry’s law is not obeyed at low pressures, and a plateau is not achieved for high pressures. Therefore, the Freundlich isotherm model lacks a thermodynamic basis, and the model is only applicable at medium pressures.^33^

The Sips isotherm model^38^ and the Toth isotherm model^39^ modify the Langmuir model by an additional parameter that describes the heterogeneity of the surface. The Sips model does not reduce to Henry’s law for low pressures, while the Toth model satisfies this condition.^32,33^ The following equations describe the Toth isotherm model as presented by Do:^28^
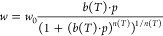
3with
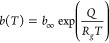
4and
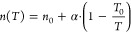
5

In eq 3, the parameters for the adsorption affinity *b*(*T*) and the heterogeneity *n*(*T*) are temperature-dependent and described by eqs 4 and 5. The parameters *w*_0_, *b*_*∞*_, *Q*, *n*_0_, and α are model parameters for a given choice of reference temperature *T*_0_. According to Do,^28^ the temperature dependence of *n* has no theoretical basis.

Shimizu and Matubayasi^40^ and Sircar^41^ analyzed the temperature dependence of the model parameters of empirical isotherm models. Both references conclude that the temperature dependence of the model parameters is not treated consistently in literature, and simplified relations for the temperature dependence can lead to erroneous results.

Temperature dependence of empirical isotherm models can also be considered based on the Clausius–Clapeyron equation.^37,42−45^ The Clausius–Clapeyron equation relies on the assumption that the enthalpy of adsorption is independent of temperature.^45^ Furthermore, the Clausius–Clapeyron equation assumes that the gas phase is an ideal gas and that the volume of the adsorbed phase is negligible.^42^

Beyond these uncertainties regarding temperature dependence, empirical isotherm models are fundamentally limited to the fluid used for parametrization. For example, the model parameters from fitting a CO_2_ isotherm cannot be used to predict the adsorption of N_2_ in the same material. Furthermore, each isotherm model corresponds to a specific shape of isotherms,^19^ and therefore, no isotherm model is generally able to describe the isotherms of any fluid and adsorbent pair. Empirical isotherm models also do not directly provide additional thermodynamic properties, such as enthalpies of adsorption, which are also relevant for process design.

Beyond empirical isotherm models and molecular simulations, adsorption behavior can be calculated by classical density functional theory (DFT).^46−49^ In recent years, a physical-based method has been developed to determine solid–fluid interactions based on one-dimensional classical density functional theory (1D-DFT) using a Helmholtz energy functional based on PC-SAFT.^50,51^ Sauer and Gross^50^ first showed the applicability of the model to calculate density profiles in slit-shaped pores. Later, Sauer and Gross^51^ enhanced the model to calculate adsorption isotherms of pure substances and mixtures for slit-shaped and cylindrical pores. The model was validated with GCMC simulations for the fluids argon, krypton, methane, and *n*-butane. Kessler et al.^52^ and Santos et al.^53^ used three-dimensional DFT (3D-DFT) to predict isotherms for 3-dimensional pore geometries. 3D-DFT uses the same inputs as GCMC simulations and can, therefore, predict adsorption properties based on the structure of solid materials.^52^ The computation of isotherms with 3D-DFT is fast compared to GCMC simulations but still orders of magnitude slower than 1D-DFT. Therefore, in our work, we focus on 1-dimensional pore geometries. The 1D-DFT model is not predictive since it relies on model parameters that describe the solid–fluid interactions. For this reason, the 1D-DFT model has to be parametrized to experimental or simulated data, similarly to empirical isotherm models.

The key benefits of the 1D-DFT model compared to empirical approaches are

1. The physical basis of the model:
  - Fluids in the porous medium and the bulk phase are consistently described by PC-SAFT, a well-established equation of state.^54^
  - The solid–fluid interactions are based on Lennard-Jones interactions that are widely used to model nonpolar intermolecular energies.
2. Extrapolation in temperature: After parametrizing the model to a single isotherm at a certain temperature, the model parameters can then be used to predict isotherms at other temperatures without the need for additional experimental or simulated data.
3. Transferability to other fluids: Due to the physical basis of the model, the parameter set describing the adsorbent obtained from the parametrization of one fluid can be transferred to other fluids. Thus, isotherms of other fluids can be calculated by using their pure component PC-SAFT parameters and standard combining rules without the need for additional experimental or simulated data.

So far, the 1D-DFT model based on PC-SAFT has only been applied to ideal, hypothetical solid materials and weakly interacting fluids such as argon, krypton, methane, and *n*-butane.^51^

In this work, we analyze the extrapolation power of the 1D-DFT model based on PC-SAFT for a wide variety of adsorbents. In particular, we study the extrapolation capability of the model parametrization from one temperature to other temperatures. We compare the results with the state-of-the-art temperature-dependent empirical isotherm models of Langmuir and Toth. These models can be parametrized if isotherms for two or more temperatures are available and interpolate between these temperatures or extrapolate outside of this temperature range.

Furthermore, we assess the transferability of parameters of the 1D-DFT model from one adsorbent/fluid-combination to another fluid. Empirical isotherm models do not permit transferring model parameters to other fluids. The extrapolation capability and parameter transferability of the 1D-DFT model have not yet been analyzed on a large set of solid materials. Due to the promising features of metal-organic frameworks (MOFs) for CO_2_ capture,^55−58^ we focus our work on this material class and the fluids CO_2_, N_2_, and CH_4_.

## 2 Methods

### 2.1 Modeling Solid–Fluid Interactions with 1D-DFT and PC-SAFT

The 1-dimensional classical density functional theory model based on PC-SAFT^51^ enables the calculation of thermodynamic equilibrium properties for the adsorption of fluids. In this work, the 1D-DFT model is used to calculate isotherms, which quantify the uptakes for a given temperature depending on the pressure. The 1D-DFT model describes the solid–fluid interactions by a pore geometry, a set of solid parameters for the adsorbent, and a set of pure component parameters characterizing the fluid within PC-SAFT. In this work, the solid adsorbent material is modeled with ideal pore geometries, either slit pores, spherical pores, or cylindrical pores. Furthermore, the adsorbent is described by 4 model parameters: pore size *r*_pore_ (radius of spherical and cylindrical pores, pore width of slit pores), segment diameter σ_*ss*_, dispersion energy parameter ε_*ss*_, and density ρ_*s*_.

Nonpolar and nonassociating fluids are described by the three pure component parameters of PC-SAFT: the number of spherical segments per chain *m*_*i*_, the segment diameter parameter σ_*ii*_, and the segment energy parameter ε_*ii*_.^54^ The parameters are used in the PC-SAFT Helmholtz energy functional, which is used to calculate the intrinsic Helmholtz energy *F*[{ρ_*i*_(**r**)}].^50^ These pure component parameters are available in literature for a wide range of species.^59^

The solid–fluid interactions are described by an external potential *V*_*i*_^ext^(**r**) where **r** is the position vector within the pore. For example, for slit pores, we describe the external potential *V*_*i*_^ext^(*z*) at a distance *z* from the wall with the Lennard-Jones 9–3 potential as
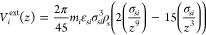
6with
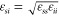
7and
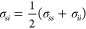
8

Expressions for the external potential of other pore geometries, such as cylindrical pores or spherical pores, can be found in Siderius and Gelb.^60^ In DFT, the grand potential Ω[{ρ_*i*_(**r**)}] combines the solid–fluid interactions via the external potential *V*_*i*_^ext^(**r**) and the fluid–fluid interactions from the intrinsic Helmholtz energy functional *F*[{ρ_*i*_(**r**)}],^61^ as
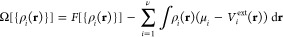
9with the chemical potential μ_*i*_ of fluid component *i*. To find an equilibrium state, the grand canonical potential functional Ω[{ρ_*i*_(**r**)}] is minimized. This minimization identifies the density profiles ρ_*i*_(**r**) for each fluid inside one pore of the adsorbent. By integrating the density profile ρ_*i*_(**r**), the uptake *w*_*i*_^pore^ within a single pore is calculated. The surface area of one pore and the internal surface area *a*_m_ per mass unit of adsorbent are used to transform the resulting uptake *w*_*i*_^pore^ per pore to an uptake *w*_*i*_ per mass unit of adsorbent. Therefore, the internal surface area *a*_m_ is an additional parameter that is necessary to describe the solid material. Adsorption isotherms are calculated by evaluating the uptake per mass unit of adsorbent at varying pressures for the same temperature.

Overall, in our work, we describe a solid material within the 1D-DFT model by 5 parameters: *r*_pore_, σ_*ss*_, ε_*ss*_, ρ_*s*_, *a*_m_. Using these 5 parameters, the pore shape type (slit, spherical, cylindrical pore shape), and the pure component parameters of PC-SAFT describing the fluid, the 1D-DFT framework calculates the isotherm of the respective adsorbent–adsorbate pair for a given temperature and pressure range. The implementation of the 1D-DFT model is provided by the open-source software FeO_s_.^62^ FeO_s_ calculates thermodynamic equilibrium properties for pure component fluids and mixtures and their adsorption on solid materials, e.g., uptakes, enthalpies of adsorption, or internal energies. For a computationally efficient evaluation of equilibrium properties, FeO_s_ implements PC-SAFT and the DFT calculations in the Rust programming language and provides a Python frontend for the analysis.

### 2.2 Parameterization of the 1D-DFT model to isotherm data

Pure component PC-SAFT parameters for common fluids are available from literature.^59^ The PC-SAFT parameters are usually parametrized to liquid densities and saturation pressures of the pure fluid. In contrast, the solid parameters of the 1D-DFT model describing the adsorbent are not yet available in the literature and have to be parametrized to experimental or simulated data. The solid parameters are used to determine the external potential *V*_*i*_^ext^(**r**), which is based on Lennard-Jones interactions between the fluid molecules and the atoms in the solid and thus independent of temperature. For this reason, parametrizations of the 1D-DFT model can intrinsically account for temperature dependence and only need to be parametrized to a single isotherm. This feature represents a major advantage since common temperature extrapolation requires at least two isotherms or the enthalpy of adsorption. An intuitive explanation is thus that the 1D-DFT model contains knowledge of the enthalpy of adsorption from the PC-SAFT description of the fluid in combination with the external potential describing its interactions with the solid.

Since the density ρ_*s*_ and the dispersion energy parameter ε_*si*_ are correlated (see eq 6), we fix the density ρ_*s*_ of the 1D-DFT model to a constant value of 0.08. Thereby, the number of model parameters to be parametrized for the adsorbent is reduced to 4. To obtain the 4 solid parameters, the 1D-DFT model is fitted to an experimental or simulated isotherm by minimizing the squared difference between the model isotherm and the isotherm data. The sum of squared errors (SSE) of the uptakes is considered as the objective function, which is frequently used for isotherm modeling.^34^ The sum of squared errors between 2 models A and B or experimental data A and model B with *m* data points *d*_*i*_ is defined by
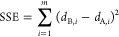
10Since the SSE is not easily interpretable, we analyze the results in Section 3 using the mean absolute relative difference (MARD), which is defined by
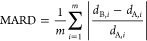
11

Given that the pore geometry representing the material is not known a priori, the parametrization is performed for all three available pore geometries (i.e., slit pores, cylindrical pores, and spherical pores). Subsequently, the pore geometry is chosen that gives the lowest objective function value in the parametrization. In this work, we show that the simplicity of ideal pores within the 1D-DFT model suffices for modeling adsorption isotherms of MOFs and fluids such as N_2_, CH_4_, or CO_2_.

## 3 Exploring the Capabilities of the 1D-DFT Model

In this section, we analyze the ability of the 1D-DFT model for temperature extrapolation and the transfer of the solid parameters determined from data of one solid–fluid pair to other fluids. For this purpose, the solid parameters need to be parametrized based on isotherm data from experiments or simulations. In our analysis, we use isotherm data generated by GCMC simulations, which allows us to study the 1D-DFT model based on an extensive, consistent data set. After parametrization, we use the obtained solid parameters to calculate isotherms at various temperatures or for fluids not used in the parametrization. For the validation of the extrapolation capabilities in temperature (see Section 3.1.1 and Section 3.2.1), we benchmark the results with empirical isotherm models from the literature. Due to their thermodynamic basis (see Section 1), we use the temperature-dependent Langmuir isotherm model^28,35^ and the Toth isotherm model.^28,39^ We choose the Langmuir isotherm model due to its simplicity and widespread use, and the Toth isotherm model because it accounts for solid heterogeneity. For the isotherms considered in our work, the temperature dependence of *n* in the Toth isotherm model is very small. Therefore, we set α = 0 (see eq 5) to reduce the number of parameters of the Toth isotherm model to 4. Temperature-dependent empirical isotherm models need data from at least two temperatures for the parametrization. In contrast, the 1D-DFT model allows for temperature extrapolation even if the model parameters are fitted solely to data at just one temperature.

The isotherm data from GCMC simulations is provided by Moubarak et al.^45^ in 2 databases:

1. For the analysis of temperature extrapolation, we use a database of isotherms for the adsorption of N_2_ and CO_2_ in 50 MOFs at 5 temperatures (298.15 K, 323.15 K, 348.15 K, 373.15 K, 398.15 K). The data at 298.15 K is also used for the analysis of the parameter transferability between N_2_ and CO_2_.
2. The parameter transferability between N_2_ and CH_4_ is analyzed with an isotherm database of the adsorption of N_2_ and CH_4_ on 406 MOFs at 298.15 K.

Both databases represent a diverse set of metals, linkers, ligands, pore sizes, and topologies.^45^ For the parametrization of both the 1D-DFT model and the empirical isotherm models, we use the nonlinear solver Knitro^63^ to identify the parameter set that minimizes the objective function.

We divide this section into two subsections: First, we investigate the nonpolar and nonassociating fluids N_2_ and CH_4_ (see Section 3.1). These fluids only have weak intermolecular interactions, are close to a spherical shape, and have negligible or no Coulombic interactions. In a second step, we validate our method for CO_2_ (see Section 3.2), which is a nonspherical molecule with a pronounced charge distribution that leads to significant Coulombic interactions between the molecule and charges in the solid structure. The 1D-DFT model does not explicitly describe Coulombic interactions and can not capture the orientation of molecules. We therefore analyze if the accuracy of the 1D-DFT model is still sufficient to describe the adsorption of CO_2_.

### 3.1 Temperature Extrapolation and Transferability for Nonpolar and Nonassociating Fluids

This section first analyzes the temperature extrapolation of the 1D-DFT model. Afterward, we investigate if a parametrization of the 1D-DFT model to N_2_ or CH_4_ isotherms can be transferred to the respective other fluid.

#### 3.1.1 Temperature Extrapolation for N_2_

**Figure 1:**
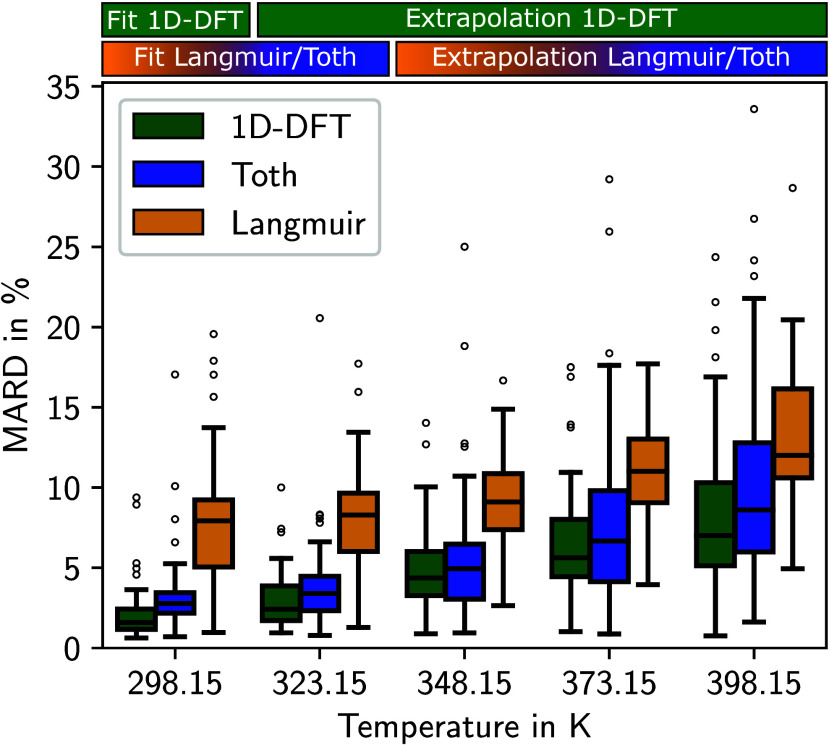
MARD of adsorption isotherms for N_2_ between 1D-DFT model and GCMC data in comparison to MARD of two empirical isotherm models (Langmuir and Toth). The empirical isotherm models are fitted to the GCMC data at 298.15 and 323.15 K. The 1D-DFT model is fitted to the GCMC data at 298.15 K. All 3 models are extrapolated to calculate the isotherms at the higher temperatures. The boxes represent the data between the first and third quartiles of the data set of 50 MOFs. The whiskers extend the boxes by 1.5 times the interquartile range.

We use pure component parameters of PC-SAFT for N_2_ from Gross and Sadowski.^54^ For each adsorbent, the 1D-DFT model is parametrized to the isotherm at 298.15 K, according to the procedure described in Section 2.2. In contrast, the Langmuir and Toth isotherm models must be parametrized to data of at least 2 temperatures to capture the temperature dependence of the isotherms. Thus, Langmuir and Toth isotherm models are parametrized to the isotherms at 298.15 and 323.15 K. The obtained parameters of the 1D-DFT model and the empirical models are used to calculate the isotherms at higher temperatures (i.e., 323.15 K for 1D-DFT, 348.15, 373.15, and 398.15 K for 1D-DFT, Toth, and Langmuir), and the results are compared to isotherm data calculated using GCMC simulations. The parametrization and validation procedure is performed for all 50 MOFs of the database of GCMC isotherms presented by Moubarak et al.^45^ The extrapolation capability of the 1D-DFT model is compared to the empirical isotherm models using the mean absolute relative deviation (MARD) for all MOFs of the database as an error criterion (Figure 1).

The 1D-DFT model proves to deliver the best extrapolation capability: isotherms calculated using the 1D-DFT model deviate less from the GCMC simulations than the ones calculated using the Langmuir or Toth isotherm model, although the 1D-DFT model is parametrized solely to isotherm data at 298.15 K. The median at the highest temperature of 398.15 K is 8% for the 1D-DFT model, which is lower than for the Toth model (9%) and the Langmuir model (12%). Moreover, the MARD of the 1D-DFT model is much lower at temperatures considered within the parametrization than for the Langmuir isotherm model and slightly lower than the Toth isotherm model. These observations indicate that the 1D-DFT model captures the shape of the isotherms used within the parametrization and the temperature dependence more accurately than the empirical isotherm models. As to be expected, the MARD increases for all 3 models with increasing temperature difference between the respective isotherm and the isotherms used in the parametrization.

**Figure 2:**
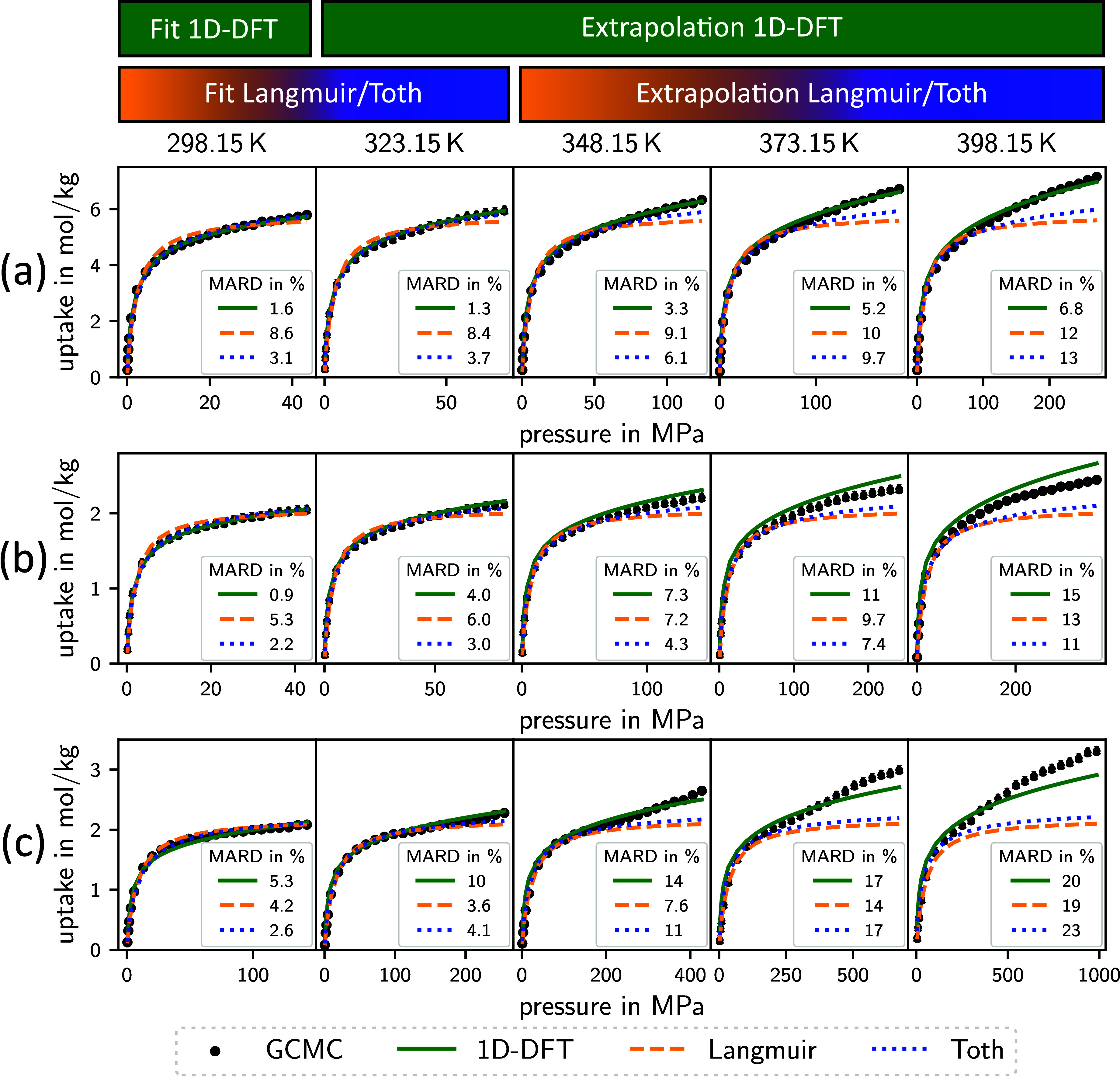
N_2_-isotherms of the MOFs SUNHIT (a), EMIYEF (b), and FIJDEI (c) at 5 temperatures calculated by GCMC, the 1D-DFT model, the Langmuir isotherm model, and the Toth isotherm model. The 1D-DFT model is fitted to the GCMC isotherm at 298.15 K. The Langmuir and Toth isotherm models are fitted to the GCMC isotherms at 298.15 and 323.15 K. The parametrizations of the 3 models are extrapolated to the isotherms at the higher temperatures. The numbers inside the boxes show the MARD of the isotherm calculated by the respective model with regard to the GCMC isotherm. Please note the changes of scale in the *x*- and *y*-axes.

We present the isotherms of the MOFs SUNHIT,^64^ EMIYEF,^65^ and FIJDEI^66^ (Cambridge Structural Database identifier^67^) as representative MOFs of the database in Figure 2 to show differences in the performance of the empirical isotherm models and the 1D-DFT model. These 3 MOFs represent MOFs with a very good performance (SUNHIT), an average performance (EMIYEF), and one of the lowest performances (FIJDEI) of the 1D-DFT model. Overall, for all 3 MOFs the 1D-DFT model results in isotherms very close to the GCMC data, demonstrating reliability in capturing isotherm behaviors at various temperatures and pressures. With an increase in temperature, the deviations between the empirical isotherm models and GCMC increases as the empirical models do not reproduce the slopes of the isotherms at high pressures. For the MOF FIJDEI (Figure 2 (c)), the 1D-DFT model is not able to fully capture the steep slope of the isotherm at high temperatures and high pressures. Nevertheless, the slope of the 1D-DFT model is closer to the slope of the GCMC data than the slope of the empirical isotherm models. Therefore, the performance of the 1D-DFT model at high pressures and high temperatures is still better than the performance of the empirical isotherm models. The errors of the 1D-DFT model for the MOF FIJDEI belong to the largest of the whole database, but still, the representation of the GCMC isotherms outperforms the empirical isotherm models. Figure 2 (b) and (c) shows that the overall representation of the isotherm can be better than the numerical values of the MARD suggest. Due to larger relative deviations at small pressures, the MARD of the Langmuir isotherm model is lower than the MARD of the 1D-DFT model while the 1D-DFT model contains smaller deviations at higher pressures. In the Supporting Information, we show the results of an adjusted error metric that reduces the impact of relative deviations at low pressures on the overall error.

In the case of MOF EMIYEF (Figure 2 (b)), the 1D-DFT model slightly overestimates the slope of the isotherm at high pressures and high temperatures. In contrast, the empirical isotherm models underestimate the slope under similar conditions and do not reproduce the shape of the isotherm as well as the 1D-DFT model. Finally, the 1D-DFT model accurately captures the slope of the isotherms of the MOF SUNHIT (Figure 2 (a)) across all temperatures and pressures. Notably, the 1D-DFT model exhibits close agreement with GCMC simulations even at 398.15 K, a temperature 100 K higher than the temperature used in the fitting process. In contrast, the empirical isotherm models do not achieve the same accuracy for the MOF SUNHIT.

In summary, for N_2_-isotherms, the 1D-DFT model has a better extrapolation capability than the Langmuir and Toth isotherm models when extrapolating a parameter set to other temperatures. The capability to extrapolate to higher temperatures is particularly notable when taking into account that the 1D-DFT model is parametrized to a single isotherm while the empirical isotherm models are parametrized to two isotherms.

#### 3.1.2 Parameter Transfer for N_2_ and CH_4_

In the 1D-DFT model, the fluid is described by its PC-SAFT pure component parameters obtained by parametrizing the PC-SAFT equation of state to saturation pressures and liquid densities of the pure substance. Thus, no adsorption data is required to parametrize a fluid, and the pure component parameters of PC-SAFT from the literature can be directly applied to calculate isotherms for fluids other than those used to parametrize the solid material. In this section, we assess the performance of the 1D-DFT model when a parameter set of the adsorbent is transferred to a fluid not used in the parametrization. Thereby, we analyze if the 1D-DFT model is able to accurately describe the solid–fluid interactions if no information on the adsorption behavior of the fluid is considered within the parametrization.

Parameter transferability cannot be benchmarked since no empirical isotherm model exists in the literature that allows for straightforward transfer of parametrizations to other fluids. Therefore, we analyze the parameter transferability of the 1D-DFT model using isotherms calculated by GCMC simulations as the benchmark. For this assessment, we use a database of 406 MOFs with isotherms of N_2_ and CH_4_ at 298.15 K calculated by GCMC simulations.^45^ The pure component parameters of PC-SAFT for N_2_ and CH_4_ are taken from Gross and Sadowski.^54^ In the first step, the 1D-DFT model is parametrized for each adsorbent to the isotherm of one fluid. In the second step, the obtained parameter set of each adsorbent is used to predict isotherms for the other fluid. This procedure is performed for both fluids, i.e., predicting CH_4_ isotherms from parameter sets obtained from N_2_ isotherms and vice versa.

**Figure 3:**
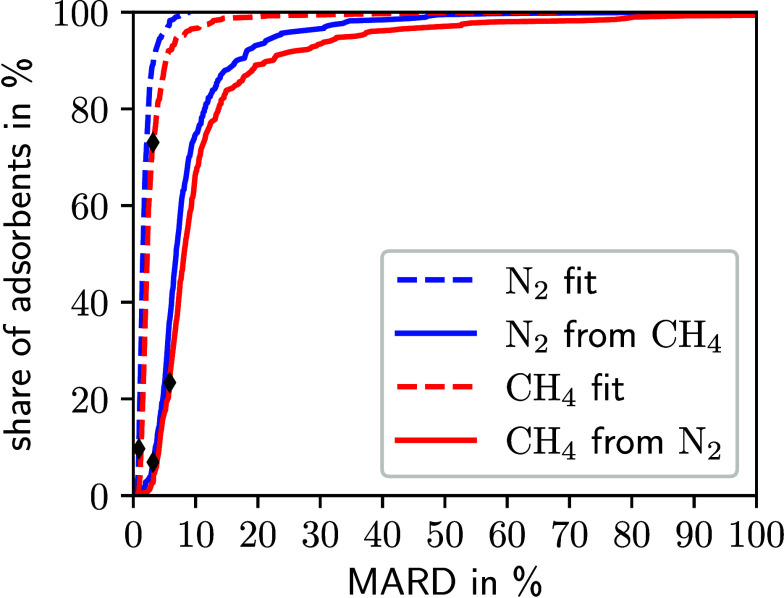
Cumulative distribution of the MARD between isotherms calculated by 1D-DFT and by GCMC for CH_4_ and N_2_ at 298.15 K. For each fluid, the cumulative distribution of the MARD is shown for the parametrization itself (dashed line) and the transfer of the parametrization to the respective other fluid (solid line). The diamond-shaped markers represent the results of the MOF GUGNON, which are shown in more detail in Figure 4.

Figure 3 shows the cumulative distribution of the MARD between the isotherms calculated by 1D-DFT for the fluid not considered in the parametrization and the isotherms obtained from GCMC simulations. For both fluids, the median of the MARD is at 10%. The MARD for N_2_ is below 20% for 93% of the MOFs. For CH_4_, 89% of the MOFs have a MARD below 20%. In total, for the 406 MOFs, we identify only 2 outliers with a MARD above 50% for a parameter transfer to N_2_ and 12 outliers for a transfer to CH_4_.

**Figure 4:**
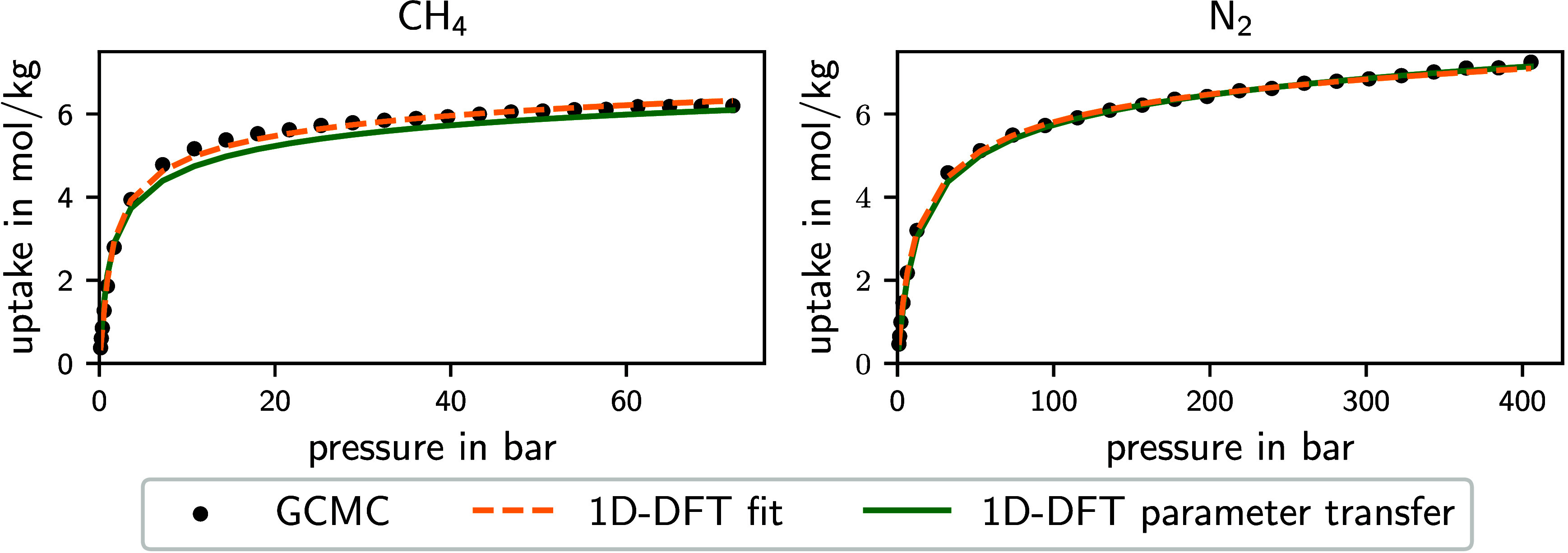
Isotherms of adsorption of CH_4_ and N_2_ on MOF GUGNON at 298.15 K. Isotherms calculated by GCMC are compared to isotherms of the 1D-DFT model fitted to the GCMC data and isotherms calculated by 1D-DFT with parameters transferred from the fit of the respective other fluid. The MARDs of the fits and the parameter transfers of this MOF are shown by diamond-shaped markers in Figure 3.

As an example, Figure 4 shows the isotherms of CH_4_ and N_2_ adsorbed on the MOF GUGNON^68^ (Cambridge Structural Database identifier^67^). For N_2_, the isotherm calculated using the parameter set of a parametrization to CH_4_ isotherm data is very close to both the GCMC data and the 1D-DFT isotherm fitted to the GCMC data with a low MARD of 3.2%. Therefore, for this material, a very good representation of the N_2_ isotherm is achieved by the parameter transfer from CH_4_. For CH_4_, the parameter transfer from N_2_ also results in a good representation of the GCMC data with only a very small offset and a MARD of 5.8%. The results highlight that a parameter transfer is possible for the fluids N_2_ and CH_4_, featuring a low average deviation and almost no model failure. For this reason, the 1D-DFT model is a powerful tool to calculate isotherms accurately and efficiently since parametrization to data of one fluid is sufficient to describe isotherms of various fluids. Therefore, the 1D-DFT model possesses the potential to reduce experimental effort or computational effort for GCMC simulations to generate adsorption property data for process design.

### 3.2 Exploring extrapolation capabilities and transferability for fluids with non-negligible Coulombic interactions

In Section 3.1, we demonstrate the high accuracy of the 1D-DFT model for temperature extrapolation and for transferring the model parameters to other fluids for the fluids N_2_ and CH_4_. However, N_2_ and CH_4_ are nonpolar, nonassociating molecules close to spherical shapes, and have negligible or no Coulombic interactions. Therefore, these molecules are expected to have similar interactions with the solid material. In contrast, CO_2_ is a nonspherical molecule with Coulombic interactions between the fluid and the solid material, which poses a challenge for the 1D-DFT model as these interactions are not explicitly described in the model. For further exploration of the 1D-DFT model, in this section, we investigate the extrapolation capabilities and parameter transferability of the 1D-DFT model for CO_2_.

#### 3.2.1 Extrapolation in Temperature for CO_2_

The parametrization and assessment procedure for CO_2_ is the same as for N_2_ in Section 3.1.1: The 1D-DFT model is parametrized to a GCMC isotherm at 298.15 K, and the Langmuir and Toth isotherm models are parametrized to GCMC isotherms at 298.15 and 323.15 K. GCMC data for 50 MOFs is taken from Moubarak et al.,^45^ and pure component parameters of PC-SAFT for CO_2_ are taken from Gross and Sadowski.^54^ We use the parametrization not considering the quadrupole moment of CO_2_ because quadrupolar interactions are neglected in the 1D-DFT model. The used parametrization of CO_2_ includes the quadrupolar interactions effectively in the dispersion parameters σ_*ii*_ and ε_*ii*_. The obtained parameter sets of the 3 models are used to calculate the isotherms at 323.15 K (1D-DFT only), 348.15, 373.15, and 398.15 K for all 50 MOFs.

**Figure 5:**
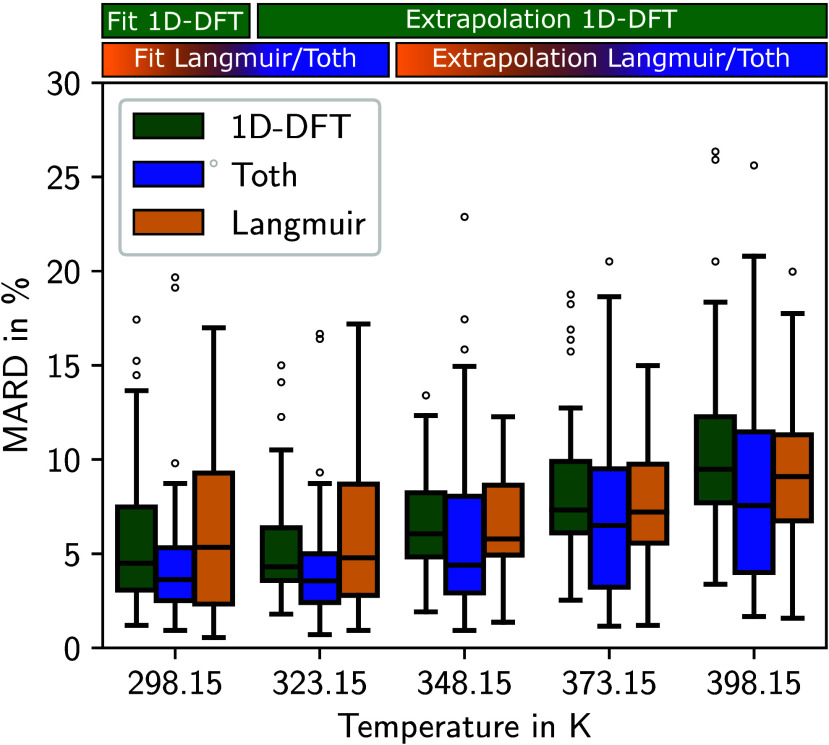
MARD of adsorption isotherms for CO_2_ between 1D-DFT model and GCMC data in comparison to MARD of two empirical isotherm models (Langmuir and Toth). The empirical isotherm models are fitted to the GCMC data at 298.15 and 323.15 K. The 1D-DFT model is fitted to the GCMC data at 298.15 K. All 3 models are extrapolated to calculate the isotherms at the higher temperatures. The boxes represent the data between the first and third quartiles of 50 MOFs. The whiskers extend the boxes by 1.5 times the interquartile range.

The 1D-DFT model and the Langmuir isotherm model show a very similar performance when extrapolating to higher temperatures (see Figure 5). For the highest temperature of 398.15 K, the median of the MARD of both models is below 10%, showing a high level of accuracy for both models. The Toth isotherm model achieves more accurate results with a lower median MARD at all temperatures.

The fit of the 1D-DFT model at 298.15 K already deviates further from the GCMC data for CO_2_ than for N_2_. Hence, the 1D-DFT model does not reproduce the shape of the CO_2_-isotherms as closely as the shape of the N_2_-isotherms. However, the deviations of the 1D-DFT model are in a similar range as the deviations of the Langmuir isotherm model for all temperatures.

In conclusion, while the interactions between fluid molecules and between the pore and the fluid molecules are simplified, the temperature extrapolation of the 1D-DFT model for CO_2_ is also accurate, albeit it does not meet the high standard of the model for N_2_. An accurate estimation of the GCMC isotherm data is possible for most MOFs, and a performance similar to the state-of-the-art Langmuir isotherm model is achievable, in particular, taking into account that the 1D-DFT model is parametrized to data at only one temperature.

#### 3.2.2 Parameter Transfer for CO_2_ and N_2_

In this section, we assess the transferability of 1D-DFT parametrizations of one fluid to the other fluid for CO_2_ and N_2_. The workflow is similar to the assessment of the transferability for N_2_ and CH_4_ (see Section 3.1.2): The solid parameters describing the adsorbent are parametrized to a GCMC isotherm of N_2_ at 298.15 K and are then used to predict the isotherm of CO_2_ at the same temperature and vice versa. GCMC data for 50 fluids is taken from Moubarak et al.^45^

**Figure 6:**
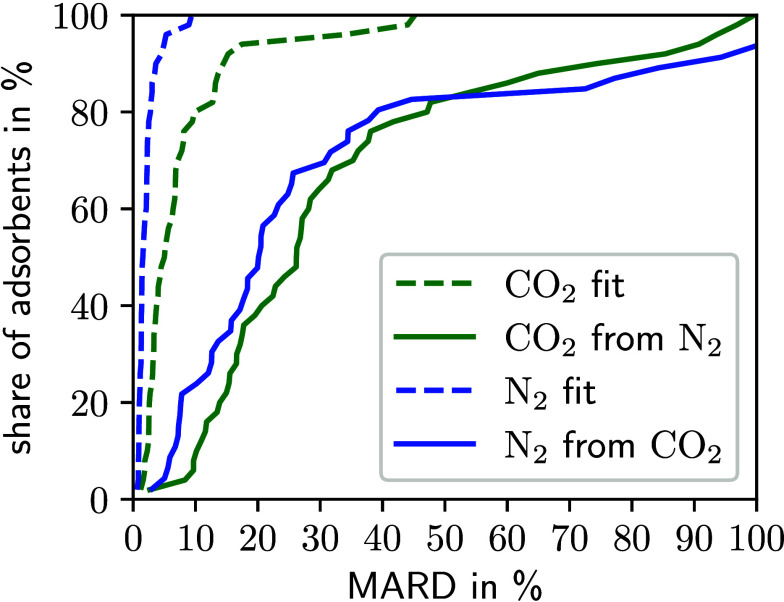
Cumulative distribution of the MARD between isotherms calculated by 1D-DFT and by GCMC for CO_2_ and N_2_. For each fluid, the cumulative distribution of the MARD is shown for the parametrization itself (dashed line) and the transfer of the parametrization to the respective other fluid (solid line).

Figure 6 shows that the MARD of 43% of the MOFs is below 20% for the parameter transfer from N_2_ to CO_2_. For this parameter transfer, the median MARD is 26%. Out of the MOFs studied, 41% exhibit a MARD below 20% when transferring parameters from CO_2_ to N_2_, with a median MARD value of 21%. Thus, an accurate reproduction of the GCMC data is possible for almost half of the materials from the MOF database. However, for some materials, the parameter transfer captures the trend of the isotherm but does not estimate the maximum uptake of the isotherm correctly, leading to high deviations. Still, only 16% of the MOFs possess deviations larger than 50%.

Results of the parameter transfer between CO_2_ and N_2_ are less favorable compared to the transfer between N_2_ and CH_4_ because for CO_2_, additional Coulombic interactions between the fluid and the solid material have to be considered that are currently not explicitly accounted for by the 1D-DFT model. However, the results still show the potential of the 1D-DFT model for complex molecules such as CO_2_, because some of the Coulombic interactions are intrinsically accounted for in the other parameters, e.g., by considering a stronger effective van der Waals interaction. In the future, improved transferability could be achieved by explicitly accounting for Coulombic interactions between the fluid molecules and the porous medium. Thereby, the accuracy of the 1D-DFT model for strongly polar molecules like water is also expected to improve.

## 4 Conclusion

We present an isotherm model to accurately determine adsorption isotherms based on classical one-dimensional density functional theory and PC-SAFT. The model parameters characterizing the adsorbents are parametrized to a single adsorption isotherm generated by experiments or molecular simulations. The so-obtained model can then be extrapolated to isotherms at other temperatures. Furthermore, the model parameters characterizing the adsorbent can be used to determine isotherms of fluids that have not been considered in the parametrization, i.e., a parameter set can be transferred to other fluids.

For the materials of the MOF database used in this work and N_2_, the presented 1D-DFT method extrapolates better in temperature than the temperature-dependent Langmuir and Toth isotherm models while requiring only data at a single temperature (i.e., half the data). For CO_2_, the accuracy of the 1D-DFT model is similar to that of the empirical isotherm models of Langmuir and Toth.

We further analyze the transferability of the solid parameter set to other fluids not used in the parametrization. If parameter sets are transferred between nonpolar and nonassociating fluids, e.g., N_2_ and CH_4_, our method determines very accurate isotherms for 90% of materials in the used database. Even though Coulombic interactions are not considered by the presented 1D-DFT model, the parameter transfer between N_2_ and CO_2_ is possible, and the trends of the isotherms are captured accurately for almost all materials.

The presented 1D-DFT isotherm model can accurately estimate isotherms for fluids and temperatures for which no experimental or simulated data is available. The only input necessary is one isotherm at one temperature for one fluid from experiments or molecular simulations and the pure component PC-SAFT parameters of the examined fluids. With these inputs, the model can be parametrized and can predict isotherms for various temperatures and various fluids. Therefore, the presented 1D-DFT model is an efficient and accurate tool for the calculation of isotherms at various conditions and a promising alternative to state-of-the-art empirical models like Langmuir or Toth. Moreover, the 1D-DFT model can calculate enthalpies of adsorption, which needs to be analyzed in detail in future work. By providing isotherms and enthalpies, the 1D-DFT model can assist in evaluating adsorption properties necessary for process models, e.g., for separation processes.

## Data Availability

The Python code developed in this work is available in the following Gitlab repository: gitlab.ethz.ch/epse/molecular-design-public/paper-adsorption-modeling-based-on-1d-dft/

## Supporting Information Available

An adjusted error metric that reduces the impact of relative deviations at low pressures on the overall error (PDF)
