# Supplementary material for: Adsorption Modeling Based on Classical Density Functional Theory and PC-SAFT: Temperature Extrapolation and Fluid Transfer
Source: Ind Eng Chem Res. 2024 Aug 1;63(32):14137–47. doi: 10.1021/acs.iecr.4c01395 (PMC11328139; doi:10.1021/acs.iecr.4c01395)
Supplement: Supplementary file 1 — ie4c01395_si_001.pdf [file ie4c01395_si_001.pdf]

Supporting Information: Adsorption modeling based on classical density functional theory and PC-SAFT: Temperature extrapolation and fluid transfer

Fabian Mayer,[†] Philipp Rehner,[†] Jan Seiler,[†] Johannes Schilling,[†] Joachim Gross,[‡] and André Bardow[*,†]

†*Energy & Process Systems Engineering, Department of Mechanical and Process Engineering, ETH Zurich, 8092 Zurich, Switzerland*

‡*Institute of Thermodynamics & Thermal Process Engineering, University of Stuttgart, 70569 Stuttgart, Germany*

E-mail: abardow@ethz.ch

S1 Relative mean absolute deviation

In Section 3.1.1, we showed that the numeric values of the error metric mean absolute relative deviation (MARD) do not always correspond to the performance observed from the plotted isotherms due to the impact of relative deviations at small pressures on the error metric. For this reason, we adjust the error metric to reduce the impact of relative deviations at small pressures. The error metric is adjusted by relating the deviations at all pressures to the maximum uptake of the GCMC isotherm data. We call the adjusted error metric relative mean absolute deviation (relMAD). The error metric relative MAD between 2 models A

and B with m data points d_i is defined by

$$\mathrm{relMAD} = \frac{1}{m}\sum_{i=1}^{m}\left|\frac{d_{\mathrm{B},i} - d_{\mathrm{A},i}}{\max\limits_i d_{\mathrm{A},i}}\right|. \tag{S1}$$

The deviations based on the relMAD of all MOFs used in our study for temperature extrapolation and N_2 are shown in Figure S1.

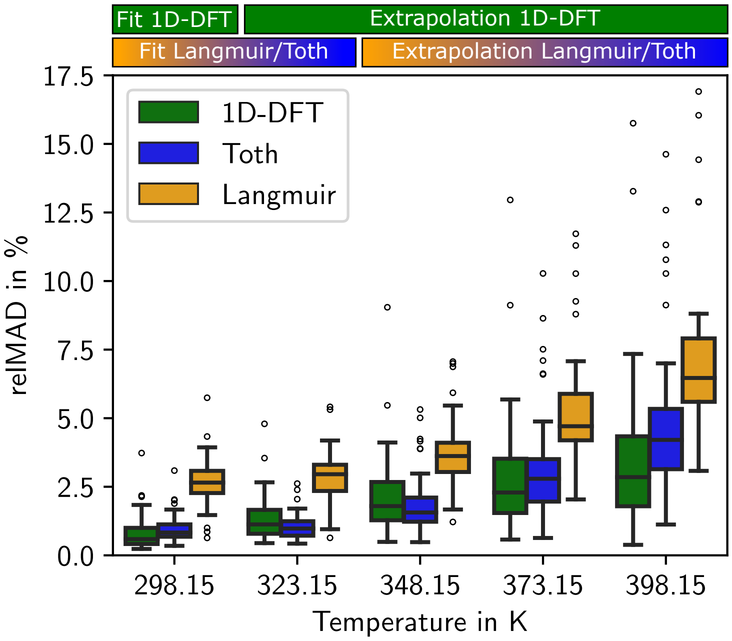

Figure S1: relMAD of adsorption isotherms for N_2 between 1D-DFT model and GCMC data in comparison to relMAD of two empirical isotherm models (Langmuir and Toth). The empirical isotherm models are fitted to the GCMC data at 298.15 K and 323.15 K. The 1D-DFT model is fitted to the GCMC data at 298.15 K. All 3 models are extrapolated to calculate the isotherms at the higher temperatures. The boxes represent the data between the first and third quartiles of the dataset of 50 MOFs. The whiskers extend the boxes by 1.5 times the interquartile range.

Qualitatively, Figure S1 is similar to Figure 1. However, the numeric values of the error metric are reduced by approximately half for all 3 models. In Table S1, the MARD and the relMAD are compared for the 3 MOFs shown in Figure 2.

Comparing the error metrics for the MOF SUNHIT, both the relMAD and the MARD of the 1D-DFT model consistently outperform both the Langmuir and Toth isotherm models across all temperature ranges, demonstrating the good performance of the 1D-DFT model.

Table S1: MARD and relMAD between N_2-isotherms calculated by GCMC and 1D-DFT, Toth, and Langmuir isotherm model. The error metric of the model with the lowest value is shown in **bold** font for each MOF and each temperature.

MOF	model	298.15 K MARD in %	298.15 K relMAD in %	323.15 K MARD in %	323.15 K relMAD in %	348.15 K MARD in %	348.15 K relMAD in %
SUNHIT	1D-DFT	**1.6**	**0.5**	**1.3**	**0.8**	**3.3**	**1.4**
	Langmuir	8.6	3.1	8.4	3.5	9.1	4.5
	Toth	3.1	0.6	3.7	1.2	6.1	2.3
EMIYEF	1D-DFT	**0.9**	**0.5**	4.0	1.8	7.3	3.5
	Langmuir	5.3	2.6	6.0	3.0	7.2	4.2
	Toth	2.2	1.1	**3.0**	**1.2**	**4.3**	**2.4**
FIJDEI	1D-DFT	5.3	2.2	10.0	2.7	14.0	**3.4**
	Langmuir	4.2	2.1	3.6	2.0	**7.6**	5.9
	Toth	**2.6**	**1.7**	**4.1**	**1.4**	10.7	5.3

MOF	model	373.15 K MARD in %	373.15 K relMAD in %	398.15 K MARD in %	398.15 K relMAD in %
SUNHIT	1D-DFT	**5.2**	**1.9**	**6.8**	**2.3**
	Langmuir	10.2	5.9	11.8	7.9
	Toth	9.7	4.1	12.8	6.2
EMIYEF	1D-DFT	10.9	5.2	14.6	**6.4**
	Langmuir	9.7	6.3	12.6	8.7
	Toth	**7.4**	**4.6**	**11.2**	7.0
FIJDEI	1D-DFT	16.9	**5.2**	19.8	**6.6**
	Langmuir	**13.8**	11.3	**19.5**	16.0
	Toth	17.0	10.3	23.2	14.6

In the case of MOF EMIYEF, the lowest value of the error metric shifts from Toth to 1D-DFT at the highest temperature of 398.15 K when shifting from MARD to relMAD. For MOF FIJDEI, the 1D-DFT model possesses the lowest relMAD for temperatures from 348.15 K and above, while the MARD is lowest for the Langmuir model at the same temperatures. The comparisons based on the relMAD correspond to the performance of the models observed in Figure 2. However, we nevertheless show the MARD in our study because the error metric is less complex and more commonly known. In conclusion, the choice of the error metric can have an impact on the results and should, therefore, be carefully selected when comparing isotherm models.
